# Are frailty and patient‐reported outcomes independent in subjects with asthma? A cross‐sectional observational study

**DOI:** 10.1111/crj.13287

**Published:** 2020-12-09

**Authors:** Masaaki Kusunose, Ryo Sanda, Mio Mori, Ayumi Narita, Koichi Nishimura

**Affiliations:** ^1^ Department of Respiratory Medicine National Center for Geriatrics and Gerontology Obu Japan; ^2^ Department of Nursing National Center for Geriatrics and Gerontology Obu Japan

**Keywords:** asthma, asthma in the elderly (AIE), frailty, health‐related quality of life (HRQOL), patient‐reported outcome (PRO)

## Abstract

**Introduction:**

This study examined the possible associations between frailty and patient‐reported outcomes (PROs) in elderly patients with asthma.

**Methods:**

Participants completed the Kihon Checklist for frailty screening as well as the following tools for measuring generic‐ and disease‐specific health‐related quality of life (HRQOL) and asthma control; the Medical Outcomes Study 36‐item short form (SF‐36), the Hyland Scale (global scale), the Asthma Quality of Life Questionnaire (AQLQ), the Asthma Control Test (ACT), and the Asthma Control Questionnaire (ACQ).

**Results:**

Of 69 consecutive outpatients with asthma, 38 (55.1%), 21 (30.4%), and 10 (14.5%) were classified as robust, pre‐frail, and frail, respectively. Eight out of 52 patients with asthma in the elderly (AIE) (>65 years old) (15.4%) were considered as being frail. The Kihon Checklist total score was significantly correlated with all the scores obtained from the SF‐36, Hyland Scale, AQLQ, ACT, and ACQ. All these scores were significantly different between groups with and without frailty. From the viewpoint of correlation coefficient, SF‐36 Physical Functioning correlated most strongly with a coefficient of −0.68 (*P* < .01), and the Hyland Scale score was second (R_S_ = −0.46, *P* < .01). The correlations between the Kihon Checklist total score and lung function parameters were weak or negative (|R_S_| < 0.35).

**Discussion:**

There were significant associations between frailty and PROs, particularly generic perception of HRQOL. Since the Kihon Checklist and PROs such as the HRQOL overlap somewhat in their evaluation of the patients’ condition, there might be some similarities in the conceptual frameworks of frailty and quality of life.

## 1 INTRODUCTION

Asthma is a common disease that is reported to affect around 339 million people globally.^1^ The prevalence of asthma in the elderly (AIE) (>65 years old) has previously been reported to be between 4% and 13%,^2^, ^3^ and the population aged 65 or older is estimated to grow from just over 520 million in 2010 to almost 1.5 billion by 2050.^4^ Elderly asthmatics are at a higher risk for morbidity and mortality than younger asthmatic patients.^5^ It has been reported that individuals identified as asthmatics showed a greater reduction in FEV_1_ with time than those who were not.^6^ It has also been stated that elderly asthmatics are vulnerable to adverse health outcomes and at risk of easily losing their quality of life (QOL) due to age‐related reductions in cognitive and physical function, and higher rates of comorbidities.^7^ Health‐related quality of life (HRQOL) is closely associated with asthma control and emergency department visits among older asthma patients as with younger asthmatics,^8^ and poorly controlled asthma is known to be a predictor of impaired HRQOL and health status.^9^ These observations indicate the importance of HRQOL as a key clinical outcome of aged patients with asthma and suggest that the clinical course of elderly patients with asthma could be worse than that of young asthmatics. An official report has cited a range of issues associated with aging that could affect both the presentation of asthma and its subsequent diagnosis and management.^7^ Based on this public health background, some review articles and recommendations focusing on AIE have been published in the last decade.^9^, ^10^ However, there have as yet only been a small number of original studies which have investigated AIE.^3^

Frailty has been recognized as a problematic condition that affects morbidity and mortality in aged people. This diagnostic approach recognizes frailty as a physical condition, purely assessed by physical measurement items and is considered to be one of the phenotype approach models. Fried et al have defined frailty as a condition fulfilling three out of five conditions comprising unintentional weight loss, self‐reported exhaustion, low grip strength, slow walking speed, and low physical activity.^11^ Although their definition has been the most widely used measure to assess frailty, there are alternative frailty diagnostic approach models such as the Frailty Index and Clinical Frailty Scale measures, known as cumulative deficit models.^12^, ^13^, ^14^ The Kihon Checklist questionnaire, one of the cumulative deficit model measures, has also been reported to be a screening tool for identifying individuals suffering from frailty. It comprises 25 items with yes/no responses, and its use in Japanese clinical practice is widespread (Supplementary File Table 1).^15^, ^16^, ^17^

Until now, as far as we are aware, there has been only one study which explores the association of frailty in subjects with asthma. The French epidemiologic cohort study recently reported that participants with asthma have increased risk of frailty.^18^ The authors hypothesized that frailty status could be associated with the severity of asthma or other clinical features including physiological factors, asthma control, or HRQOL. The purpose of the present study was to examine the prevalence of frailty and to clarify its relationship with features of asthma in a clinical setting.

## 2 MATERIALS AND METHODS

### 2.1 Participants

Sixty‐nine consecutive patients with stable asthma from the outpatient clinic of the Department of Respiratory Medicine of the National Center for Geriatrics and Gerontology (NCGG) volunteered to participate between March 2016 and July 2018. The entry criteria for adults with asthma were as follows: a history of asthma symptoms, an unchanged treatment protocol for more than 4 weeks, no exacerbations of their asthma over the preceding 6 weeks, regular attendance at our establishment for more than 6 months, and absence of comorbidities severely affecting clinical condition. Although there was no age limit for enrollment, the majority of participants were expected to be outpatients with AIE, that is, over 65 years old since the establishing aim of our institute was for geriatric diseases. So that patients with chronic obstructive pulmonary disease (COPD) were excluded, those enrolled had either never smoked or if they were current or former smokers, had a maximum forced expiratory volume in 1 second (FEV_1_)/forced vital capacity (FVC) ratio exceeding 0.7 on an earlier measurement. All of the participants gave written informed consent. In accordance with the Global Strategy for Asthma Management and Prevention (2019 update) issued by the Global Initiative for Asthma (GINA),^19^ the treatments were broadly grouped into the following five steps: Step 1: absence of daily inhaled corticosteroid (ICS); Step 2: daily low‐dose ICS; Step3: low‐dose ICS‐long‐acting beta_2_‐agonist (LABA); Step 4: medium‐dose ICS‐LABA; and Step 5: high‐dose ICS‐LABA.

### 2.2 Measurement

The following tests were conducted for every eligible patient on the same day: clinical and functional measures including serum immunoglobulin E (IgE) level as well as pulmonary function tests, assessment of frailty, and patient‐reported outcome (PRO) measurement. All participants underwent assessments of the levels of serum total IgE and specific IgE to house dust, dermatophagoides pteronyssinus and dermatophagoides farinae. If any of these IgE levels exceeded the normal upper value, we defined the patient’s IgE elevation as positive. Pulmonary function testing was performed after participants had been asked to cease inhalation of corticosteroids and bronchodilators prior to the study. Participants underwent post‐bronchodilator spirometry (CHESTAC‐8800; Chest, Tokyo, Japan), residual volume (RV) measured by the closed‐circuit helium method, and diffusing capacity for carbon monoxide (DL_CO_) measured by the single‐breath technique in accordance with the standards of the European Respiratory Society and American Thoracic SocietyTask Force in 2005.^20^ The predicted values for FEV_1_ and vital capacity were calculated according to the proposal from the Japanese Respiratory Society.^21^

### 2.3 Frailty assessment

The Kihon Checklist is a self‐administered checklist comprising 25 items which are yes/no questions (Supplementary File Table 1).^15^, ^17^ The 25 questions include seven categories: instrumental (three items), social activities of daily living (four items), physical strength (five items), nutritional status (two items), oral function (three items), cognitive status (three items), and depression risk (five items). Among these items, body mass index (BMI) which is the subject of question No. 12 was calculated using values obtained at the same time as the pulmonary function tests, so this question was not self‐reported here. The Kihon Checklist total score is the sum of 25 answers, and ranges from 0 (no frailty) to 25 (severe frailty). We categorized patients’ frailty status into robust (0‐3), pre‐frail (4‐7), and frail (8‐25), respectively.^17^

### 2.4 Patient‐reported outcome measurement

All participants completed a self‐administered booklet which included the Japanese versions of the following PRO measures; the Asthma Quality of Life Questionnaire (AQLQ), the Asthma Control Test (ACT), the Asthma Control Questionnaire (ACQ), the Medical Outcomes Study 36‐item short form (SF‐36), and the Hyland Scale.^22^, ^23^, ^24^, ^25^, ^26^, ^27^, ^28^ The completed booklets were all checked by a member of the research team (K.N.) to avoid the possibility of missed items.

Assessments of the degree of asthma control were conducted using the ACT and ACQ.^27^, ^28^ The ACT is a measure for identifying poorly controlled asthma with a 4‐week recall period, and includes five items each with a 5‐point scale.^28^ The ACT is scored from 5 to 25 with lower scores indicating worse control. The ACQ is a tool for evaluating adequacy of asthma control. In this study, five items were evaluated over a 1‐week period.^27^ Patients responded to each item scaled from 0 (no impairment) to 6 (maximum impairment) and the total score was calculated as the mean of the sum of five items.

The AQLQ is a validated measure evaluating disease‐specific QOL comprising 32 items with a 14‐day recall period.^22^ The AQLQ has 4 domains: Symptom (11 items), Activity Limitation (12 items), Emotional Function (5 items), and Environmental Exposure (4 items). Each item is scored from 1 to 7, and the mean of each domain is the AQLQ score. A higher score indicates a better QOL.

To assess generic health status, we used version 2 of the Japanese SF‐36 composed of eight subscales (Physical Functioning, Role Physical, Bodily Pain, General Health, Vitality, Social Functioning, Role Emotional, and Mental Health).^23^, ^25^, ^26^ The SF‐36 with norm‐based scorings are arranged to mean scores of 50 and a standard deviation of 10 in the Japanese general population. Higher scores reflect better health conditions. The Japanese version of the Hyland Scale was also used to measure global health. Its scores range from 0 to 100, where 0 = “might as well be dead” and 100 = “perfect quality of life”.^24^

### 2.5 Statistical methods

All results are expressed as means ± standard deviation (SD). A *P* value of less than .05 was considered to be statistically significant. Score distributions obtained from the tools were evaluated by histograms and the Shapiro‐Wilk test. Relationships between two sets of data were analyzed by Spearman’s rank correlation tests. Between‐group differences were examined using analysis of variance, and Bonferroni tests were conducted post hoc when these differences were found to be significant. A stepwise regression analysis was used to identify potential predictors for the Kihon Checklist total score and to account for the effect on that score. All statistical analyses were performed using IBM SPSS Statistics for Windows v.19.

## 3 RESULTS

A total of 69 consecutive adults (34 men) with asthma, and a wide range of FEV_1_ (68.8 ± 20.2%pred) were studied (Table 1). The mean age of the participants was 69.4 ± 10.8 years and 52 of these (75.4%) were diagnosed with AIE, defined as aged more than 65 years. Thirty‐eight (55.0%) participants were considered to have atopy and 36 (52.2%) were never smokers. Inhaled corticosteroids (ICS) were prescribed to all patients and inhaled long‐acting beta agonists (LABAs) were prescribed to 59 (85.5%). Six participants (8.7%) were also receiving long‐acting inhaled anticholinergics.

**TABLE 1:** Baseline characteristics in 69 patients with asthma and Spearman’s rank correlation coefficients with the Kihon Checklist total score

		Mean ± SD	Range	R_S_
Age	years	69.4 ± 10.8	41‐88	0.39**
BMI	kg/m^2^	24.2 ± 3.7	16.1‐32.5	–
FVC	Liters	2.89 ± 0.83	1.41‐5.52	−0.34**
FEV_1_	Liters	2.10 ± 0.62	1.04‐3.67	−0.34**
FEV_1_/FVC	%	72.9 ± 8.3	45.0‐90.7	–
TLC	Liters	4.57 ± 1.08	2.56‐7.76	−0.27*
DL_CO_	mL/min/mm Hg	14.60 ± 5.01	4.50‐27.05	−0.31**
PaO_2_	mm Hg	83.0 ± 9.7	58.8‐101.3	–
ACT	(5‐25)	21.6 ± 3.5	11‐25	−0.45***
ACQ	(0‐6)	0.48 ± 0.74	0.00‐3.60	0.33**
AQLQ^a^ Symptom	(1‐7)	6.38 ± 0.66	4.75‐7.00	−0.32**
AQLQ^a^ Activity limitation	(1‐7)	6.18 ± 0.86	3.73‐7.00	−0.50***
AQLQ^a^ Emotional function	(1‐7)	6.22 ± 0.94	3.60‐7.00	−0.24*
AQLQ^a^ Environmental exposure	(1‐7)	6.54 ± 0.67	4.50‐7.00	−0.29**
SF‐36^b^ Physical functioning	(0‐100)	46.2 ± 11.9	3.7‐57.8	−0.68***
SF‐36^b^ Role physical	(0‐100)	48.9 ± 10.5	12.5‐55.7	−0.41**
SF‐36^b^ Bodily pain	(0‐100)	51.6 ± 10.8	22.4‐61.7	−0.39**
SF‐36^b^ General health	(0‐100)	49.1 ± 8.9	29.8‐69.8	−0.42***
SF‐36^b^ Vitality	(0‐100)	54.3 ± 8.3	33.8‐69.1	−0.34**
SF‐36^b^ Social functioning	(0‐100)	52.1 ± 8.5	24.8‐57.0	−0.41**
SF‐36^b^ Role emotional	(0‐100)	50.8 ± 10.0	6.1‐56.1	−0.42***
SF‐36^b^ Mental health	(0‐100)	55.0 ± 8.8	35.7‐65.2	−0.34**
Hyland scale score	(0‐100)	73.9 ± 1.6	35‐100	−0.46**

Notes

Missing values of correlation coefficients indicate no statistically significant relationship. The numbers in parentheses denote possible score ranges.

Abbreviations: ACQ, Asthma Control Questionnaire; ACT, Asthma Control Test; AQLQ, Asthma Quality of Life Questionnaire; BMI, body mass index; DL_CO_, diffusing capacity for carbon monoxide; FEV_1_, forced expiratory volume in 1 second; FVC, forced vital capacity; PaO_2_, partial pressure of arterial oxygen; SF‐36, Short Form 36‐Item; TLC, total lung capacity.

^a^ *n* = 65;

^b^ *n* = 66;

* *P* < .05;

** *P* < .01;

*** *P* < .001.

**FIGURE 1:**
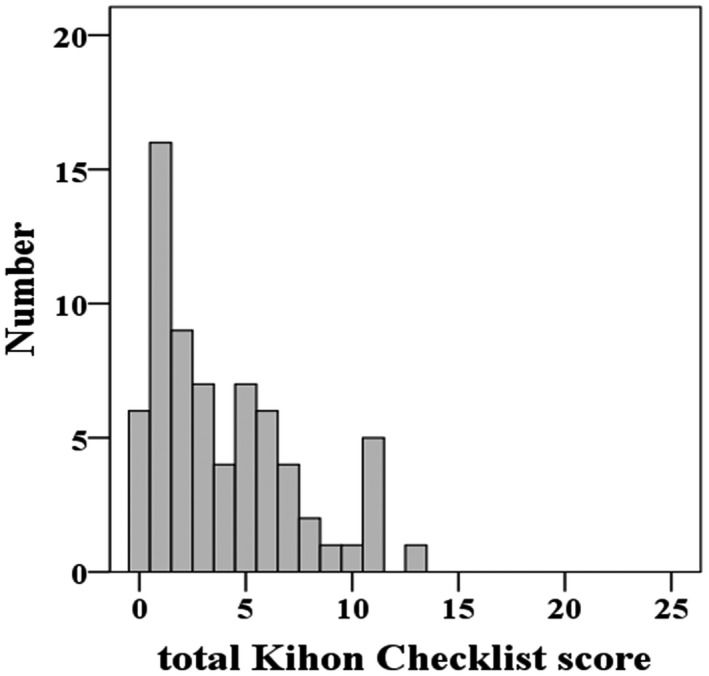
Frequency distribution histogram of the Kihon Checklist total score

The frequency distribution of the Kihon Checklist total scores is depicted in Figure 1, showing a non‐normal score distribution (Shapiro‐Wilk test, *P* < .001). Depending on the Kihon Checklist total score, participants were classified into the following three groups: 38 (55.1%) robust, 21 (30.4%) pre‐frail, and 10 (14.5%) frail. In addition, 8 out of the 52 participants with AIE (15.4%) were considered to be frail.

**FIGURE 2:**
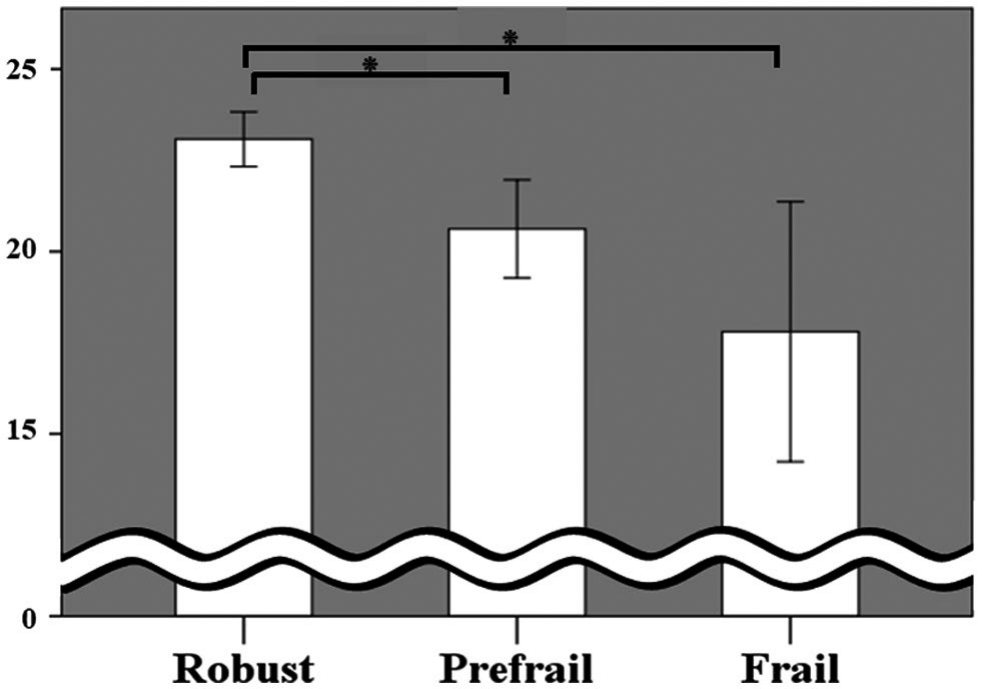
Comparison of asthma control measured by the Asthma Control Test (ACT) among groups of robust, pre‐frail, and frail. Asterisks (*) show statistically significant differences (*P* < .05)

**FIGURE 3:**
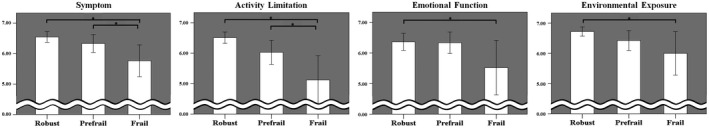
Comparison of disease‐specific health‐related quality of life measured by four domains of the Asthma Quality of Life Questionnaire (AQLQ) among groups of robust, pre‐frail, and frail. Asterisks (*) show statistically significant differences (*P* < .05)

**FIGURE 4:**
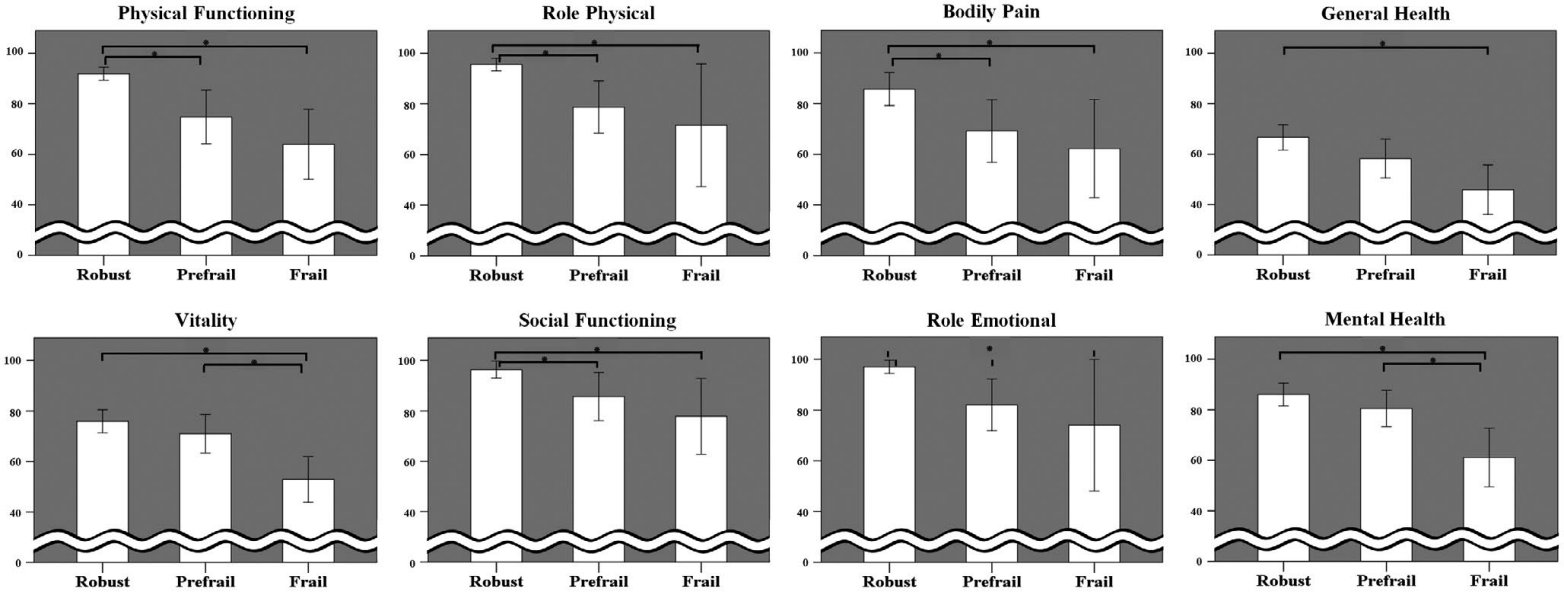
Comparison of generic health‐related quality of life measured by eight subscales of SF‐36 among groups of robust, pre‐frail, and frail. Asterisks (*) show statistically significant differences (*P* < .05)

We then compared the differences between groups with and without frailty as categorized by the Kihon Checklist total score (Figures 2, 3, 4 and Supplementary File Table 2).While physiological parameters such as the results of pulmonary function testing showed no significant between‐group differences (Supplementary File Table 2), there were significant between‐group differences in the scores obtained from tools to measure asthma control (Figure 2), disease‐specific (Figure 3), and generic (Figure 4) HRQOL. All scores obtained from the ACQ, AQLQ, and SF‐36 were significantly different between the robust and frail groups. Some scores were significantly different between the robust and pre‐frail groups, and others between the pre‐frail and frail groups.

The correlations between the Kihon Checklist total scores and clinical and physiological factors are listed in Table 1. Age had statistically significant correlations with the Kihon Checklist total score (R_S_ = 0.39, *P* < .01). With respect to pulmonary function testing, FVC (L) (R_S_ = −0.34, *P* = .01), FEV_1_ (L) (R_S_ = −0.34, *P* < .01), TLC (L) (R_S_ = −0.27, *P* = .03), DL_CO_ (ml/min/mm Hg) (R_S_ = −0.31, *P* = .01), and DL_CO_ (% pred) (R_S_ = −0.26, *P* = .03) had only weak correlations with the Kihon Checklist total score. As for PROs, the Kihon Checklist total scores had moderate correlations with all of the domains of the AQLQ score (R_S_ = −0.25 to −0.50, *P* < .01 or *P* < .05), the ACT score (R_S_ = −0.45, *P* < .01), and the Hyland Scale score (R_S_ = −0.46, *P* < .01). All SF‐36 subscale scores were also moderately or weakly correlated with the Kihon Checklist total scores. From the viewpoint of correlation coefficient, SF‐36 Physical Functioning correlated most strongly with a coefficient of −0.68 (*P* < .01), and the Hyland Scale score was second (R_S_ = −0.46, *P* < .01). Table 2 shows the comparison of frequency of frailty, clinical indices, and scores obtained from the Kihon Checklist and patient‐reported outcome measures between the treatment steps provided by the GINA. All participants with frailty are categorized as being in Step 5, which is the severe asthma group.

**TABLE 2:** Comparison of frequency of frailty, clinical indices, and scores obtained from the Kihon Checklist and patient‐reported outcome measures between the treatment steps currently provided by the Global Initiative for Asthma (GINA)

		STEP 1+2	STEP 3+4	STEP 5
		*N* = 8	*N* = 12	*N* = 49
Frail	Frequency	*N* = 0 (0%)	*N* = 0 (0%)	*N* = 10 (20%)
The Kihon Checklist total score	(0‐25)	2.8 ± 2.0	2.3 ± 2.1	4.6 ± 3.6
Age	Years	70.9 ± 6.0	61.6 ± 15.3	71.1 ± 9.0^b^
BMI	kg/m^2^	24.8 ± 4.2	24.0 ± 3.5	24.2 ± 3.7
FVC	Liters	2.73 ± 0.53	3.40 ± 0.92	2.79 ± 0.79
FEV_1_	Liters	2.10 ± 0.43	2.55 ± 0.72	1.99 ± 0.55^b^
FEV_1_/FVC	%	77.1 ± 5.5	74.9 ± 6.3	71.8 ± 8.7
TLC	Liters	4.31 ± 0.83	5.06 ± 0.98	4.50 ± 1.10
DL_CO_	mL/min/mm Hg	12.82 ± 5.07	17.45 ± 5.23	14.20 ± 4.61
PaO_2_	mm Hg	83.2 ± 7.4	86.2 ± 10.1	82.1 ± 9.6
ACT	(5‐25)	22.6 ± 3.0	22.6 ± 2.7	21.1 ± 3.6
ACQ	(0‐6)	0.2 ± 0.2	0.1 ± 0.2	0.6 ± 0.8
AQLQ^1^ Symptom	(1‐7)	6.8 ± 0.4	6.8 ± 0.1	6.2 ± 0.7^c^
AQLQ^1^ Activity limitation	(1‐7)	6.6 ± 0.6	6.6 ± 0.6	6.0 ± 0.9
AQLQ^1^ Emotional function	(1‐7)	6.7 ± 0.4	6.8 ± 0.3	6.0 ± 1.0^b^
AQLQ^1^ Environmental exposure	(1‐7)	6.9 ± 0.2	6.8 ± 0.4	6.4 ± 0.7
SF‐36^2^ Physical functioning	(0‐100)	88.6 ± 8.9	88.2 ± 23.5	80.6 ± 17.6
SF‐36^2^ Role physical	(0‐100)	91.4 ± 10.8	96.0 ± 5.5	84.4 ± 22.1
SF‐36^2^ Bodily pain	(0‐100)	94.8 ± 9.4	88.5 ± 18.1	71.9 ± 24.6^a^
SF‐36^2^ General health	(0‐100)	70.5 ± 14.9	58.9 ± 16.9	60.2 ± 16.2
SF‐36^2^ Vitality	(0‐100)	84.4 ± 10.4	71.6 ± 11.4	68.9 ± 16.5^a^
SF‐36^2^ Social functioning	(0‐100)	95.3 ± 8.7	94.3 ± 9.8	88.8 ± 18.3
SF‐36^2^ Role emotional	(0‐100)	90.6 ± 12.8	97.0 ± 7.3	87.4 ± 22.1
SF‐36^2^ Mental health	(0‐100)	89.4 ± 8.5	81.8 ± 11.1	79.4 ± 17.7
Hyland scale score	(0‐100)	78.8 ± 15.2	81.3 ± 12.3	71.3 ± 15.5

Abbreviations: ACQ, the Asthma Control Questionnaire; ACT, the Asthma Control Test; AQLQ, the asthma quality of life questionnaire; DL_CO_, diffusing capacity for carbon monoxide; FEV_1_, forced expiratory volume in 1 second; FVC, forced vital capacity; PaO_2_, partial pressure of arterial oxygen; SF‐36, Short Form 36‐Item; TLC, total lung capacity.

^a^ *P* < .05 versus STEP 1+2;

^b^ *P* < .05 versus STEP 3+4;

^c^ *P* < .05 versus STEP 1+2 and STEP 3+4;

^1^ *n* = 65;

^2^ *n* = 66.

Stepwise multivariate regression analysis was then performed to identify those variables that predicted the Kihon Checklist total score. We chose several independent variables on the basis of our presumption that they were related to the clinical course of asthma and frailty: age (years), serum Ig E level, airflow limitation (FEV_1_,L), asthma control (ACT score), and generic HRQOL (SF‐36 PF) as explanatory variables. Using a multiple linear regression model with the backward stepwise method, the only significant predictive variable of the Kihon Checklist total score was SF‐36 Physical Functioning (*β* = −0.62, *P* < .01). However this variable explained only 38.6% of the variance.

## 4 DISCUSSION

As far as we are aware, only one epidemiological study of frailty in asthma patients has been published hitherto,^18^ and this is the second report on the prevalence of frailty and its close relationship with HRQOL in patients with asthma. The present study showed that the prevalence of frailty was 14.5% in 69participantswith asthma, whose mean age was 69.4 years, including 15.4% of the 52 with AIE. A systematic review and meta‐analysis by Marangoni et al reported that the prevalence of frailty in individuals with COPD was 20%,^29^ and our own previous study which was conducted in a similar manner at the same site also showed that 21.5% of outpatients with COPD were considered as beingfrail.^30^ Therefore, compared with COPD, prevalence of frailty appeared to be somewhat lower in asthma.

We explored the cross‐sectional relationship between frailty status using the Kihon Checklist questionnaire and various clinical indices in patients with asthma. Significant correlations were found between patient‐reported outcome measurements and the Kihon Checklist total score, and there were statistically significant between‐group differences between the robust and the pre‐frail or frail groups. So far, the Kihon Checklist has been considered to reflect cumulative deficits in relation to physical, psychological, and social conditions of elderly patients. From the viewpoint of correlation coefficients between the Kihon Checklist total score and scores obtained from tools for PRO measure, SF‐36 Physical Functioning correlated most strongly with a coefficient of −0.68. Since the Hyland Scale score was second, the Kihon Checklist total score appears to be closer to the generic perception related to HRQOL. In other words, since the Kihon Checklist and PROs such as the HRQOL overlap somewhat in their evaluation of the patients’ condition, there might be some similarities in the conceptual frameworks of frailty and QOL. Another point is that there is the possibility that the factors that determine poor health also determine what frailty is, at least in the case of how it is defined in the Kihon checklist.

As the Kihon Checklist is in common use throughout Japan,^15^, ^17^ we aimed to investigate which variables contribute to the Kihon Checklist total score by stepwise multiple regression analyses, but cumulative coefficients of determination were relatively low. Over half of the variance is unexplained, suggesting that the contributory factors we selected were not necessarily appropriate and that other variables could better explain the total Kihon Checklist score. The result of this approach is similar to that of our previous study conducted on participants with COPD.^30^

Our study contained some limitations, most of which relate to its design. First, the present single‐center study was limited by the small number of participants with AIE even though asthma is considered to be one of the common disorders. This study may include selection bias because we recruited those patients who were able to regularly visit our establishment. It is possible that we unintentionally excluded an appreciable number of those patients who ignored respiratory symptoms and were unaware of having asthma, or those unable to visit our clinic on a regular basis due to their heavy physical burden. However, it did contain all of the patients with asthma who were able to enroll during the study period since the mission of our institute is to provide health services for the aged population. Taking this possible population bias into consideration, generalization of these results may be uncertain. Second, we focused on correlational analysis, which is not optimal for identifying the potential causes of a phenomenon.

In conclusion, this is the first study assessing the prevalence of frailty in patients with asthma, especially AIE. It was shown that frailty screened by the Kihon Checklist might be more closely associated with PROs relating to generic HRQOL than lung function in patients with asthma. It may be speculated that there are some similarities in the conceptual frameworks of frailty and QOL.

## CONFLICT OF INTEREST

There are no conflict of interest to declare with respect to any of the authors.

## ETHICS

Ethics approval for this study was granted by the Institutional Ethics Committee of the National Center for Geriatrics and Gerontology (No. 1194).

## AUTHOR CONTRIBUTIONS

MK helped to analyze the results, perform the statistical analysis, and write the initial draft. RS, MM, and AN facilitated the conduct of the study and collection of data. KN helped with the study concept and design, and with interpreting and editing the manuscript. The authors have all read and approved the final draft.

## Supporting information

Table S1

Table S2

## Data Availability

The datasets generated during and/or analyzed during the current study are available from the corresponding author on reasonable request.

## References

[1] 2018 The Global Asthma Network . The Global Asthma Report 2018. Auckland, New Zealand: The Global Asthma Network; 2018.

[2] Kim Y‐K , Kim S‐H , Tak Y‐J , et al. High prevalence of current asthma and active smoking effect among the elderly. Clin Exp Allergy. 2002;32(12):1706‐1712. 12653160 10.1046/j.1365-2222.2002.01524.x

[3] Enright PL , McClelland RL , Newman AB , Gottlieb DJ , Lebowitz MD . Underdiagnosis and undertreatment of asthma in the elderly. Cardiovascular Health Study Research Group. Chest. 1999;116(3):603‐613. 10492260 10.1378/chest.116.3.603

[4] National Institute on Aging, National Institutes of Health . Global health and aging. NIH Publication no. 11‐7737; 2011.

[5] Dunn RM , Busse PJ , Wechsler ME . Asthma in the elderly and late‐onset adult asthma. Allergy. 2018;73(2):284‐294. 28722758 10.1111/all.13258

[6] Lange P , Parner J , Vestbo J , Schnohr P , Jensen G . A 15‐year follow‐up study of ventilatory function in adults with asthma. N Engl J Med. 1998;339(17):1194‐1200. 9780339 10.1056/NEJM199810223391703

[7] Skloot GS , Busse PJ , Braman SS , et al. An Official American Thoracic Society workshop report: evaluation and management of asthma in the elderly. Ann Am Thorac Soc. 2016;13(11):2064‐2077. 27831798 10.1513/AnnalsATS.201608-658ST PMC5466180

[8] Kannan JA , Bernstein DI , Bernstein CK , et al. Significant predictors of poor quality of life in older asthmatics. Ann Allergy Asthma Immunol. 2015;115(3):198‐204. 26208758 10.1016/j.anai.2015.06.021 PMC4567431

[9] Chen H , Gould MK , Blanc PD , et al. Asthma control, severity, and quality of life: quantifying the effect of uncontrolled disease. J Allergy Clin Immunol. 2007;120(2):396‐402. 17561244 10.1016/j.jaci.2007.04.040

[10] Hanania NA , King MJ , Braman SS , et al. Asthma in the elderly: Current understanding and future research needs–a report of a National Institute on Aging (NIA) workshop. J Allergy Clin Immunol. 2011;128(3 Suppl):S4‐S24. 21872730 10.1016/j.jaci.2011.06.048 PMC3164961

[11] Fried LP , Tangen CM , Walston J , et al. Frailty in older adults: evidence for a phenotype. J Gerontol A Biol Sci Med Sci. 2001;56(3):M146‐M157. 11253156 10.1093/gerona/56.3.m146

[12] Mitnitski AB , Mogilner AJ , Rockwood K . Accumulation of deficits as a proxy measure of aging. Sci World J. 2001;1:323‐336. 10.1100/tsw.2001.58 PMC6084020 12806071

[13] Rockwood K , Song X , MacKnight C , et al. A global clinical measure of fitness and frailty in elderly people. CMAJ. 2005;173(5):489‐495. 16129869 10.1503/cmaj.050051 PMC1188185

[14] Brummel NE , Bell SP , Girard TD , et al. Frailty and subsequent disability and mortality among patients with critical illness. Am J Respir Crit Care Med. 2017;196(1):64‐72. 27922747 10.1164/rccm.201605-0939OC PMC5519959

[15] Arai H , Satake S . English translation of the Kihon Checklist. Geriatr Gerontol Int. 2015;15(4):518‐519. 25828791 10.1111/ggi.12397

[16] Dent E , Kowal P , Hoogendijk EO . Frailty measurement in research and clinical practice: a review. Eur J Intern Med. 2016;31:3‐10. 27039014 10.1016/j.ejim.2016.03.007

[17] Satake S , Senda K , Hong Y‐J , et al. Validity of the Kihon Checklist for assessing frailty status. Geriatr Gerontol Int. 2016;16(6):709‐715. 26171645 10.1111/ggi.12543

[18] Landré B , Nadif R , Goldberg M , et al. Asthma is associated with frailty among community‐dwelling adults: the GAZEL cohort. BMJ Open Respir Res. 2020;7(1):e000526. 10.1136/bmjresp-2019-000526 PMC7047496 32066563

[19] Global Initiative for Asthma . Global Strategy for Asthma Management and Prevention. Fontana, WI: Global Initiative for Asthma (GINA); 2019.

[20] Miller MR , Hankinson J , Brusasco V , et al. Standardisation of spirometry. Eur Respir J. 2005;26(2):319‐338. 16055882 10.1183/09031936.05.00034805

[21] Sasaki H , Nakamura M , Kida K , Kambe M , Takahashi K , Fujimura M . Reference values for spirogram and blood gas analysis in Japanese adults. J Jpn Respir Soc. 2001;39(5):S1‐S17.

[22] Juniper EF , Guyatt GH , Epstein RS , Ferrie PJ , Jaeschke R , Hiller TK . Evaluation of impairment of health related quality of life in asthma: development of a questionnaire for use in clinical trials. Thorax. 1992;47(2):76‐83. 1549827 10.1136/thx.47.2.76 PMC463574

[23] Ware JE Jr , Sherbourne CD . The MOS 36‐item short‐form health survey (SF‐36). I. Conceptual framework and item selection. Med Care. 1992;30(6):473‐483. 1593914

[24] Hyland ME , Sodergren SC . Development of a new type of global quality of life scale, and comparison of performance and preference for 12 global scales. Qual Life Res. 1996;5(5):469‐480. 8973126 10.1007/BF00540019

[25] Fukuhara S , Bito S , Green J , Hsiao A , Kurokawa K . Translation, adaptation, and validation of the SF‐36 Health Survey for use in Japan. J Clin Epidemiol. 1998;51(11):1037‐1044. 9817121 10.1016/s0895-4356(98)00095-x

[26] Fukuhara S , Ware JE Jr , Kosinski M , Wada S , Gandek B . Psychometric and clinical tests of validity of the Japanese SF‐36 Health Survey. J Clin Epidemiol. 1998;51(11):1045‐1053. 9817122 10.1016/s0895-4356(98)00096-1

[27] Juniper EF , O'Byrne PM , Guyatt GH , Ferrie PJ , King DR . Development and validation of a questionnaire to measure asthma control. Eur Respir J. 1999;14(4):902‐907. 10573240 10.1034/j.1399-3003.1999.14d29.x

[28] Nathan RA , Sorkness CA , Kosinski M , et al. Development of the asthma control test: a survey for assessing asthma control. J Allergy Clin Immunol. 2004;113(1):59‐65. 14713908 10.1016/j.jaci.2003.09.008

[29] Marengoni A , Vetrano DL , Manes‐Gravina E , Bernabei R , Onder G , Palmer K . The relationship between COPD and frailty: a systematic review and meta‐analysis of observational studies. Chest. 2018;154(1):21‐40. 29477493 10.1016/j.chest.2018.02.014

[30] Kusunose M , Oga T , Nakamura S , Hasegawa Y , Nishimura K . Frailty and patient‐reported outcomes in subjects with chronic obstructive pulmonary disease: are they independent entities? BMJ Open Respir Res. 2017;4(1):e000196. 10.1136/bmjresp-2017-000196 PMC5531303 28883929

