# Supplementary material for: Are frailty and patient‐reported outcomes independent in subjects with asthma? A cross‐sectional observational study
Source: Clin Respir J. 2020 Dec 9;15(2):216–24. doi: 10.1111/crj.13287 (PMC7983993; doi:10.1111/crj.13287)
Supplement: Supplementary file 1 — Table S1 [file CRJ-15-216-s001.pdf]

Supplementary Table 1 The Kihon Checklist.

No.	Questions	Answer	
1	Do you go out by bus or train by yourself?	☐0. YES	☐1. NO
2	Do you go shopping to buy daily necessities by yourself?	☐0. YES	☐1. NO
3	Do you manage your own deposits and savings at the bank?	☐0. YES	☐1. NO
4	Do you sometimes visit your friends?	☐0. YES	☐1. NO
5	Do you turn to your family or friends for advice?	☐0. YES	☐1. NO
6	Do you normally climb stairs without using handrail or wall for support?	☐0. YES	☐1. NO
7	Do you normally stand up from a chair without any aids?	☐0. YES	☐1. NO
8	Do you normally walk continuously for 15 minutes?	☐0. YES	☐1. NO
9	Have you experienced a fall in the past year?	☐0. YES	☐1. NO
10	Do you have a fear of falling while walking?	☐0. YES	☐1. NO
11	Have you lost 2 kg or more in the past 6 months?	☐0. YES	☐1. NO
12	Height: cm, weight: kg, BMI: kg/m2 If BMI is less than 18.5, this item is scored.	☐0. YES	☐1. NO
13	Do you have any difficulties eating tough foods compared to 6 months ago?	☐0. YES	☐1. NO
14	Have you choked on your tea or soup recently?	☐0. YES	☐1. NO
15	Do you often experience having a dry mouth?	☐0. YES	☐1. NO
16	Do you go out at least once a week?	☐0. YES	☐1. NO
17	Do you go out less frequently compared to last year?	☐0. YES	☐1. NO
18	Do your family or your friends point out your memory loss? e.g.“You ask the same question over and	☐0. YES	☐1. NO
19	Do you make a call by looking up phone numbers?	☐0. YES	☐1. NO
20	Do you find yourself not knowing today’s date?	☐0. YES	☐1. NO
21	In the last 2 weeks have you felt a lack of fulfilment in your daily life?	☐0. YES	☐1. NO
22	In the last 2 weeks have you felt a lack of joy when doing the things you used to enjoy?	☐0. YES	☐1. NO
23	In the last 2 weeks have you felt difficulty in doing what you could do easily before?	☐0. YES	☐1. NO
24	In the last 2 weeks have you felt helpless?	☐0. YES	☐1. NO
25	In the last 2 weeks have you felt tired without a reason?	☐0. YES	☐1. NO

BMI, body mass index
