# Supplementary material for: Are frailty and patient‐reported outcomes independent in subjects with asthma? A cross‐sectional observational study
Source: Clin Respir J. 2020 Dec 9;15(2):216–24. doi: 10.1111/crj.13287 (PMC7983993; doi:10.1111/crj.13287)
Supplement: Supplementary file 2 — Table S2 [file CRJ-15-216-s002.docx]

| Supplementary File. Table 2. Comparison of clinical indices and scores obtained from patient-reported outcome measures between robust, pre-frail and frail groups classified by the Kihon Checklist total score. | | | | |
| --- | --- | --- | --- | --- |
|  |  | Robust | Pre-frail | Frail |
|  |  | (n=38) | (n=21) | (n=10) |
| Age | years | 66.4 ± 10.5 | 71.7 ± 10.9 | 76.0 ± 8.2^a^ |
| BMI | kg/m^2^ | 23.9 ± 3.8 | 25.5 ± 3.6 | 22.8 ± 3.6 |
| FVC | Liters | 3.00 ± 0.80 | 2.99 ± 0.93 | 2.30 ± 0.44 |
| FEV_1_ | Liters | 1.58 ± 0.12 | 2.06 ± 0.44 | 1.41 ± 0.22 |
| FEV_1_/FVC | % | 67.2 ± 13.8 | 71.2 ± 1.9 | 76.6 ± 6.7 |
| TLC | Liters | 4.67 ± 1.04 | 4.75 ± 1.23 | 3.83 ± 0.56 |
| DL_CO_ | mL/min/mmHg | 10.80 ± 1.59 | 13.79 ± 5.11 | 8.50 ± 2.06 |
| PaO_2_ | mmHg | 83.1 ± 9.6 | 80.5 ± 10.8 | 87.5 ± 5.8 |
| ACQ | (0-6) | 0.20 ± 0.40 | 1.20 ± 2.08 | 1.70 ± 0.14^a^ |
| Hyland Scale score | (0-100) | 75.0 ± 8.2 | 73.3 ± 22.5^a^ | 57.5 ± 10.6^a^ |
| a: p<0.05 vs. robust group; FVC, forced vital capacity; FEV_1_, forced expiratory volume in 1 second; TLC, total lung capacity; DL_CO_, diffusing capacity for carbon monoxide; PaO_2_, partial pressure of arterial oxygen; ACQ, the Asthma Control Questionnaire. | | | | |
|  |  |  |  |  |
|  |  |  |  |  |
|  |  |  |  |  |
